# Screen Use, Child Development and Family Involvement in Children Aged 2–7 Years: A Systematic Review

**DOI:** 10.1111/jpc.70533

**Published:** 2026-08-11

**Authors:** Mónica Gomariz‐Martínez, Pedro Gil‐Madrona, Luisa Losada‐Puente

**Affiliations:** ^1^ Faculty of Education University of Castilla‐La Mancha Albacete Castilla‐La Mancha Spain; ^2^ Faculty of Educational Sciences University of A Coruña A Coruna Spain

**Keywords:** child development, children, families, screen time, systematic review

## Abstract

**Introduction:**

This study aimed to explore the effects of screen time on the development of children and the mediating role of families, following the PRISMA method.

**Methods:**

Searches were conducted in Web of Science, Scopus and ERIC databases between 2020 and 2025. Eighteen articles met inclusion criteria: participants aged 2–7 years; specified screen time duration; reported associations with child development; and examination of family‐related factors.

**Results:**

Nearly 62% of studies reported screen use exceeding recommended limits. Socio‐emotional development was the most frequently examined domain, followed by cognitive, language and motor/physical domains. Excessive screen time was associated with poorer attention and working memory, hyperactivity and impulsivity symptoms, language comprehension and production deficits, emotional regulation difficulties and reduced family interactions. Parental modelling, supervision, joint viewing, educational content use and shared reading emerged as moderating factors.

**Discussion:**

Overall, children's digital device use was influenced by family practices and educational use.

## 1 Introduction

Early exposure of children to digital devices has generated particular concern among health and education experts. Technological advances have fostered the emergence and widespread availability of a wide range of electronic devices in households [1]. Televisions, laptops, tablets and smartphones, among others, are increasingly integrated into children's everyday environments from very early ages (American Academy of Paediatrics Council on Communications and Media, [2]). The total amount of time devoted to the use of these screen‐based devices, the so‐called *screen time*, includes both passive and active content consumption, as well as digital interactions [3].

Many organisations have established specific guidelines regarding appropriate screen exposure time according to age, aiming at minimising associated risks and promoting healthy lifestyles during childhood. In 2016, the AAP warned about the impact of digital media on children's health and recommended completely avoiding screen media use before 18–24 months of age, as well as limiting screen time to about of 1 h per day for children aged 2–5 years. Priority should be given to high‐quality content, supervised by adults, to promote learning. Also, they recommended avoiding screen use for at least 1 h before bedtime. More recent guidance from the AAP reflects a shift away from simple screen time limits toward a broader understanding of children's digital media use within digital ecosystems, emphasising content quality, context and adult co‐engagement rather than fixed time thresholds [4]. Similarly, the World Health Organization (WHO) published its recommendations in 2019, advising against screen exposure before the age of 2 years and recommending no more than 1 h/day for children aged 2–4 years and encourage to replace sedentary time by active play and caregiver‐child interaction [5]. Moreover, recent studies point out a widespread tendency to exceed these recommended limits. A study conducted by UNICEF in 2021 showed that the average age at which children access their first phone is 10.96 years (SD = 1.65) [6]. The International Early Learning and Child Well‐Being Study indicated that 83% of 5‐year‐old children in Estonia, the United Kingdom and the United States used digital devices at least one a week, with 42% doing so on a daily basis (Organisation for Economic Co‐operation and Development, [7]). More recently, the OECD report *How's life for children in the Digital Age?* [8] highlights that digital environments present both opportunities (e.g., for learning, creativity and social connection) and risks (e.g., impacts on sleep, physical activity and mental health) and that such impacts vary with the type of digital activity and contextual factors. Patterns of use and personal, family and environmental characteristics influence outcomes.

To gain an in‐depth understanding of the potential effects of screen use in child development, it is necessary to draw on developmental theories [9, 10, 11]. At a cognitive level, early childhood is marked by the emergence of executive functions (memory, inhibitory control and cognitive flexibility), alongside social interactions and the acquisition of language. Several studies point to a complex relationship between screen exposure and cognitive development, given its multifactorial nature and its dependence on factors such as the type and quality of content, the child's active or passive engagement during use, the family context and individual child characteristics [12, 13, 14, 15]. About physical and motor development, factors such as increased sedentary behaviour and sleep disturbances have been associated with prolonged screen use [16], as well as deficits in motor skill development [17]. In addition, frequent use of touchscreen devices has been shown to negatively affect manual skills [18]. At the socioemotional level, problematic and excessive use of digital devices during early childhood has been documented [19], for instance, to soothe tantrums, manage challenging behaviours or serve as a distraction. Such practices may hinder the development of emotional self‐regulation [20, 21] and lead to a reduction in social skills [22].

Family mediation plays a key role in the supervision, regulation and interpretation of the content that children consume on digital devices [23]. Greater parental modelling, screen use during meals and the use of screens as a distractor, emotional regulator or reward are associated with higher levels of screen use among children, whereas the establishment of rules regarding screen use is associated with healthier usage patterns [24]. This increased screen use among children is, in turn, related to low socioeconomic status, passive parenting behaviours and parental psychological distress [25].

## 2 Research Question and Objectives

Excessive digital media use among preschool‐aged children is not an isolated phenomenon but is influenced by multiple factors, including the family and social environment. Moreover, inequalities in adults' access to digital media and quality education may limit their ability to engage in effective mediation practices. This limitation hinders the implementation of strategies for guidance, supervision and regulation of children's screen use, thereby contributing to prolonged screen exposure during early childhood. From this context emerges the following research question: What effect does excessive screen use have on children's holistic development during early childhood and what role do families play in this relationship?

This research question was formulated using the PICO method (population, intervention, comparison, outcome) [26] (Table 1).

**TABLE 1:** Research question developed using PICO strategy.

Population	Children aged 2–7 years, without special educational needs and their families.
Intervention	Time of exposure to screen‐based devices and the type of family involvement.
Comparison	Children with lower screen exposure time.
Result	Associations and effects of screen time on overall development (cognitive, language, physical and motor and socioemotional development), as well as influence or moderating effect of family involvement.

*Note:* This table presents the research question defined for this review. Adapted from *PICO Worksheet and Search Strategy* by Miller [26].

To address this question, the main objective of the study was to examine the scientific literature regarding the relationship between excessive screen use and the overall development of children aged 2–7 years, as well as to examine the role of families in managing this relationship. The specific objectives were the following:

- To descriptively analyse the scientific literature published over the last 5 years on the effects of screen use on child development in children aged 2–7 years.
- To identify associations between excessive screen exposure time and the different dimensions of child development.
- To examine the effects of screen use on child development between the ages of 2 and 7 years.
- To explore family involvement in children's screen use and its influence on development in early childhood.

## 3 Method

The study followed the *Preferred Reporting Items for Systematic Reviews and Meta‐Analyses* (PRISMA) guidelines [27]. Prior to conducting the review, a search was carried out in the DARE (Database of Abstracts of Reviews of Effects) and PROSPERO (International Prospective Register of Systematic Reviews) databases using the descriptors ‘screentime’ AND ‘child’ AND ‘development’, with the aim of identifying previous systematic reviews addressing similar topics. No results were found in DARE; however, PROSPERO identified systematic reviews focused on screen exposure time that addressed only a single developmental dimension or populations different from those considered in the present review [28, 29, 30]. This systematic review was not prospectively registered. Study materials are available in the Open Science Framework: https://osf.io/35mtz/overview.

The authors declare that the research presented in this manuscript complies with the ethical principles established by the University of A Coruña. No human subjects were involved, as this is a systematic review.

### 3.1 Inclusion and Exclusion Criteria

Peer‐reviewed articles published in English or Spanish were included. The search was limited to publications from January 2020 to December 2025 to update existing evidence following the review by Streegan et al. [31], which covered studies published between 2000 and March 2020 and supported reducing screen exposure time among children aged 0–9 years in line with AAP recommendations. No systematic reviews published after 2020 were identified that specifically addressed this topic while incorporating the role of families in regulating children's screen use.

Studies included if participants were children aged 2–7 years without special educational needs; screen time duration and measurement instruments were specified; associations between screen time and child developmental outcomes were reported; and family involvement in children's screen use was examined. Studies were excluded if they included other age groups without disaggregated results for the 2–7‐year‐olds; focused exclusively on perceptions of parents or teachers without direct data on child development; were systematic reviews, meta‐analyses or intervention proposals; or addressed screen use during COVID‐19, as this period reflected exceptional circumstances.

### 3.2 Search Strategy

The search was conducted on the Web of Science (WOS) and Scopus, as internationally recognised databases and in ERIC, a key database in the field of education. Searches were carried out between June 2025 and January 2026. The final search was conducted on January 15, 2026. The search strategy was based on the review by Streegan et al. [31], with the addition of a specific block addressing family involvement. Descriptors were organised into four thematic blocks: target population, screen use, child development and family involvement and combined using Boolean operators. The terms employed were: (‘child*’ OR ‘infant’ OR ‘preschool’ OR ‘school‐aged’) AND (‘screen time’ OR ‘screen use’ OR ‘screen exposure’ OR ‘tablet’ OR ‘mobile’ OR ‘videogame’ OR ‘televis*’ OR ‘screen‐based media’) AND (‘neurodevelopment’ OR ‘developmental outcomes’ OR ‘development’ OR ‘cognitive’ OR ‘executive functions’ OR ‘academic’ OR ‘vocabulary’ OR ‘language’ OR ‘attention’ OR ‘motor’ OR ‘motor skills’ OR ‘emotional’ OR ‘socio‐emotional’) AND (‘education’ OR ‘school’ OR ‘family’ OR ‘parents’ OR ‘management’ OR ‘reduce’ OR ‘prevent’ OR ‘guideline’).

The search yielded 7.469 records. After duplicate removal, 5.445 articles remained. Screening of titles, abstracts and keywords led to the exclusion of 5.062 records. Two reviewers independently screened titles and abstracts. The remaining 383 full‐text articles were assessed for eligibility, resulting in the exclusion of 367 studies. The final sample comprised 18 articles included in the review (Figure 1).

**FIGURE 1:**
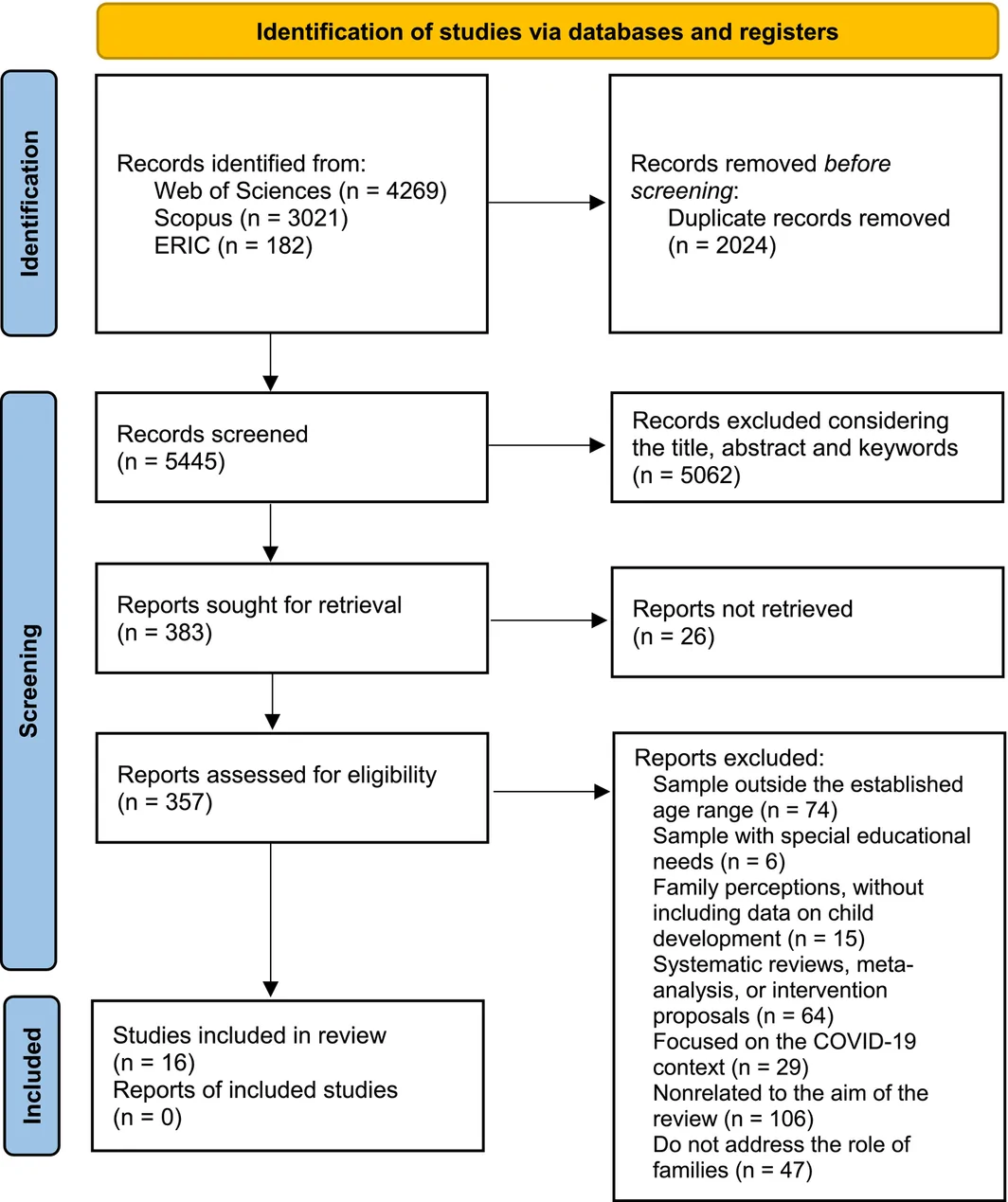
PRISMA flow diagram. *Source:* Page et al. [27]. This work is licensed under CC BY 4.0. To view a copy of this licence, visit https://creativecommons.org/licenses/by/4.0/.

## 4 Data Extraction and Analysis

Data from the included studies were extracted and organised in a Microsoft Excel spreadsheet by one reviewer. Recorded outcome variables included the developmental domain addressed, author(s) and year of publication, country, sample size and age range, data collection instruments and key findings. Attention was paid to the role of families in each study. The methodological quality of the included studies was assessed using the Joanna Briggs Institute critical appraisal tools. Two reviewers independently evaluated each study. Most studies showed moderate methodological quality, primarily due to the reliance on parent‐reported measures of children's screen exposure and the limited identification or control of potential confounding factors (see Table S1). To ensure the accuracy of the dataset, the extracted information was subsequently reviewed and verified by one additional reviewer. Any discrepancies identified during this process were resolved through discussion until consensus was reached.

This process enabled descriptive analyses of the extracted variables. Subsequently, a manual qualitative analysis was conducted, without the use of specific software, to identify and systematise the main themes in relation to the review objectives. Based on this thematic analysis, findings were organised into two main analytical categories with corresponding subcategories: the impact of screen time on child development (cognitive, language, physical and motor and socioemotional development); and family involvement in screen use and its influence on child development (parental supervision practices, mental health and modelling, quality of parent–child interactions and emotional bonding and shared reading and screen use). Due to the heterogeneity of the studies, effect size measures were not synthesised quantitatively.

## 5 Results

### 5.1 Descriptive Characteristics of the Studies

Eighteen scientific articles published between 2020 and 2025 were included, with 2024 standing out with five publications. Most studies were conducted in Asia (seven studies: two in Turkey and in Thailand and one in China, India and Thailand), followed by Europe (six studies: two in Finland and one in Denmark, Germany, Hungary, Portugal), North America (two articles, in Canada and United States), Oceania (two articles: in Australia and New Zeeland) and South America (one study in Brasil).

Quantitative cross‐sectional designs predominated, with only two longitudinal studies identified [32, 33]. Sample size ranged from 44 participants [34] to 31.125 participants [35]. Most studies focused on children aged 2–5 years, with little representation of children aged 6–7 years [33, 36, 37]. Data were primarily collected through parent‐reported questionnaires and surveys, combined with standardised assessments of cognitive, language, motor and/or socio‐emotional development. Screen time and parenting practices were measured using both study‐specific instruments [34, 38, 39] and validated tools [40, 41, 42], with frequent use of the Five‐to‐Fifteen questionnaire and the Behaviour Rating Inventory of Executive Function‐Preschool version (BRIEF‐P).

Descriptive analysis showed that 61.15% of the studies (*n* = 11) reported screen use exceeding the recommended maximum of 1 h/day by the Council on Communications and Media [2]. Regarding the dependent variable (child development), studies focused on one or more dimensions of child development and how these are affected using various types of electronic devices. Overall, the most researched dimension was socio‐emotional development, with 34.38%, followed by cognitive development (28.12%), language development (25%) and physical and motor development (12.5%) (Figure 2).

**FIGURE 2:**
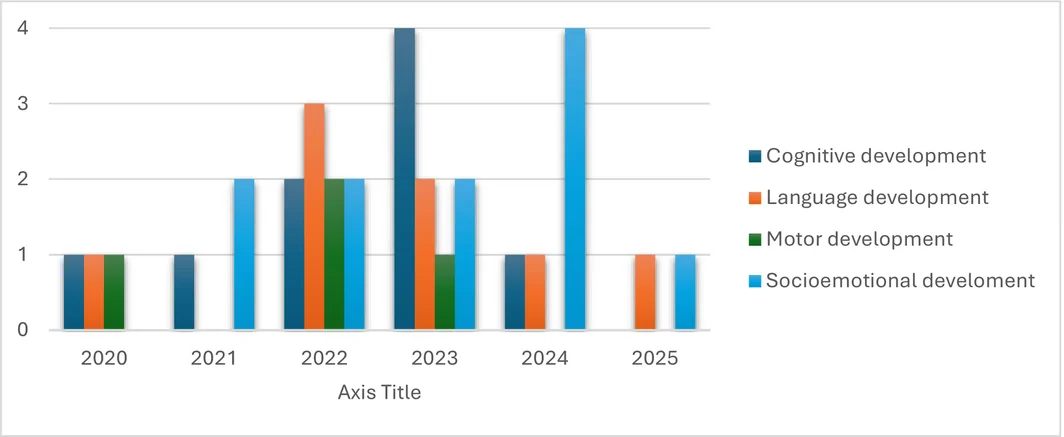
Distribution of the studied developmental dimensions by year of publication. This graph represents the dimensions of development studied in articles by year of publication. Some studies analyse several dimensions of development, so the total sum exceeds *n* = 17. Own elaboration.

### 5.2 Impact of Screen Time on Child Development

Nine studies examined associations between screen time and *children's cognitive development*, many of them finding cognitive difficulties in children with longer screen time. For instance, Niiranen et al. [43] reported attention and concentration difficulties and symptoms of hyperactivity and impulsivity in 5‐year‐old children with high screen time (*p* = 0.03). Similarly, Wu et al. [44] found that exceeding the recommended screen time was associated with attention difficulties (*β* = 7.20, 95% CI [1.50,12.91], *p* < 0.05). Rai et al. [34] reported negative associations between video/movie viewing and working memory (*r =* −0.42, *p* < 0.01) and Schwarzer et al. [45] found lower cognitive scores among children aged 2–5 years who spent < 1 h/day in front of a screen (*b =* −10.96, 95% CI [−18.69, −3.24], *p* < 0.001). In contrast, Danet et al. [32] reported no association between total screen time and executive functions.

Content type was also relevant. Rai et al. [34] and Nobre et al. [42] reported positive associations between educational screen use and inhibitory control (*r* = 0.33, *p* = 0.01) and cognitive development (*r* = 0.23, *p* = 0.04), respectively. However, Danet et al. [32] found weaker executive functioning among children using educational apps (aOR = 1.36, 95% CI [1.09, 1.70], *p* < 0.05), with no associations for streaming video or inappropriate app use. Finally, Wannapaschaiyong et al. [37] reported that working memory impairment was significantly associated with prolonged screen time to low‐quality content (aOR = 6.97, 95% CI [1.37, 35.40], *p* < 0.001).

Across eight studies, daily screen time of 1 h or more was associated with poorer language development. For example, Rayce et al. [35] found that daily use between 1 and 2 h increased the likelihood of language comprehension difficulties by 30% (OR = 1.30, 95% CI [1.19, 1.41], *p <* 0.001), rising to 42% when use exceeded 2 h (OR = 1.42, 95% CI [1.08, 1.88], *p < 0*.05). For expressive language, the risk increased by 19% (aOR = 1.19, 95% CI [1.03, 1.37], *p < 0*.05) and 46% (aOR = 1.46, 95% CI [1.10, 1.93], *p* < 0.01), respectively. Similarly, Schwarzer et al. [45] report significantly lower language development percentiles when exposure exceeds 1 h/day (*b =* −12.88, 95% CI [−20.19, −5.57], *p* < 0.01). Mustonen et al. [41] showed that each additional 30 min/day of unsupervised screen use was associated with declines in expressive lexical skills (*r* = −0.22, *p* < 0.05) and overall language skills (*r* = −0.18, *p* < 0.05) with unsupervised screen time predicting both expressive vocabulary (*B* = −0.08, *p* < 0.05) and total language skills (*B* = −0.13, *p* < 0.05). Gath et al. [46] also observed lower scores in language production with increased screen time (*r* = −0.35, *p* < 0.01), while the association with comprehension was weaker and non‐significant.

Content quality also appeared relevant. Rai et al. [34] and Nobre et al. [42] reported positive associations between educational or high‐quality digital media use and vocabulary and language development (*r* = 0.38, *p* = 0.02 and *r* = 0.40, *p* < 0.001). In contrast, Cadime et al. [47] found no direct associations between overall screen time and expressive vocabulary or syntactic complexity in children aged 2.5–3 years, but observed that higher screen time was associated with less frequent shared reading (*β* = −0.11, *p* < 0.10), the latter being the main predictor of language development (*β* = 0.30, *p* < 0.001). Maternal education was also related to lower screen time (*β* = −0.27, *p* < 0.001) and higher levels of shared reading (*β* = 0.20, *p* < 0.001), suggesting an indirect effect of screen use on language through family interactions. Chaibal and Chaiyakul [48] found no significant relationships between frequent smartphone and tablet use and language development in children aged 2–5 years, identifying only signs (9.42%) of possible developmental delays.

Only four studies examined the relationship between digital device use and *physical and motor development* in early childhood, with findings varying by the context of use. Chaibal and Chaiyakul [48] identified suspected delays in fine‐adaptive motor (32.94%) and gross motor development (2.35%) among children aged 2–5 years with frequent smartphone and tablet use. Greater device use was significantly associated with poorer gross motor development (*χ* ^2^ = 6.657, *p* = 0.04), while no association was observed for fine‐adaptive motor development. In contrast, Nobre et al. [42] reported a weak but significant positive association between the quality of digital media use and fine motor development (*r* = 0.22, *p = 0*.04), with no association for gross motor development. Two studies also examined sleep outcomes, reporting more sleep‐related problems among children exceeding recommended screen time. Wu et al. [44] pointed out an average increase of 9.87 points on sleep difficulty scale (*β* = 9.87, 95% CI [1.34, 18.40], *p* < 0.05), while Cadime et al. [47] reported a significant association between higher screen time and sleep outcomes (*ρ* = −0.11, *p* < 0.05).

High screen time was consistently associated with poorer socioemotional outcomes in early childhood. Niiranen et al. [43] found that high screen time in 5‐year‐old children was associated with increased internalising (e.g., anxiety) and externalising symptoms (e.g., aggressiveness, impulsivity) (*p* < 0.001). Mallawaarachchi et al. [33] reported no cross‐sectional associations between mobile device use and internalising or externalising behaviours; however, among toddlers, a lower proportion of passive viewing (i.e., interactive app use) predicted higher externalising behaviours 1 year later (*R* ^*2*^ = 0.08, *p* < 0.001), with no associations in preschoolers. Liu et al. [40] found a significant negative association between electronic media use and prosocial behaviour (*β* = −0.19, 95% CI [−0.27, −0.10], *p < 0*.001). Similarly, Oflu et al. [38] reported higher emotional lability and negativity among children using screens for 4 h/day or more compared with those with lower exposure (38.8% vs. 21.8%; *p* < 0.01), as well as among children without parental co‐viewing (39.1% vs. 25.0%; *p* = 0.03). Consistently, Wannapaschaiyong et al. [37] found that unsupervised device use (< 50% parental co‐viewing) was strongly associated with executive function impairments, including deficits in impulse control (aOR = 11.12, 95% CI [2.69, 45.96], *p* < 0.05) and emotional regulation (aOR = 17.87, 95% CI [1.75, 182.18], *p* < 0.05). Poor supervision also increased the likelihood of excessive screen use (OR = 3.7, 95% CI [1.3, 10.8], *p <* 0.05) and social skills deficits (OR = 15.3, 95% CI [1.9, 121.2], *p* < 0.05) [49].

Regarding response to screen restriction, Sari and Yalçın [39] found that 44.4% of children reacted by crying, whereas 55.6% engaged in alternative activities. Positive reactions were more likely among children with < 1 h/day of screen use (OR = 2.65, 95% CI [1.22–5.78], *p =* 0.01), lower parental screen use (OR = 2.01, 95% CI [1.11–3.62], *p =* 0.02) and more supportive home literacy environments. In contrast, Schwarzer et al. [45] reported significantly lower socioemotional skills among children with > 1 h/day of screen time (*b =* −7.80, 95% CI [−15.45, −0.14], *p* < 0.05) and Chaibal and Chaiyakul [48] identified suspected delays in personal‐social development (11.76%) among children with high screen use.

### 5.3 Family Factors Shaping Children Digital Media Use and Development

Family involvement in children's digital device use between ages 2–7 years emerged as a key factor across most reviewed studies. Three main forms of family involvement were identified: parental supervision practices (*n* = 2), parental modelling (*n* = 7) and joint use of devices for reading or co‐viewing (*n* = 5).

Regarding *parental supervision*, John et al. [49] found that unintentional supervision of screen use was associated with higher odds of attention (OR = 3.2, 95% CI [1.3, 8.2], *p* < 0.05), cognitive (OR = 4.1, 95% CI [1.3, 13.3], *p* < 0.05) and social deficits (OR = 15.3, 95% CI [1.9, 121.2], *p* < 0.05). Similarly, Oflu et al. [38] observed that the absence of parental supervision (aOR = 0.42, 95% CI [0.21, 0.82], *p* = 0.01) and the child being primarily cared for by the mother (aOR = 16.88, 95% CI [2.85, 99.96], *p* < 0.01) were associated with screen use of 4 h or more per day. Higher emotional lability was also observed among children with high screen exposure without parental co‐viewing, with 39.1% falling into the highest quartile compared with 25.0% of those whose screen use was parent‐accompanied (*p* = 0.03).

Evidence on *parental modelling* consistently showed that parents' digital device use influenced children's usage patterns. Mustonen et al. [41] reported that maternal screen time was negatively associated with children's expressive vocabulary (*β* = −0.02, *p* < 0.01) and overall language abilities (*β* = −0.04, *p* < 0.01), with a 120‐min daily increase in maternal screen use corresponding to an estimated 2.8‐point decrease in expressive vocabulary and a 4.4‐point decrease in overall language scores. Chaibal and Chaiyakul [48] found positive correlations between children's device use and screen use by mothers (*r* = 0.29, *p* = 0.02) and other family members (*r* = 0.70, *p* < 0.05), a pattern replicated by Schwarzer et al. [45], who reported higher child screen use when maternal use exceeded 5 h/day (OR = 3.86, 95% CI [1.80–8.26], *p* < 0.01). In contrast, Sari and Yalçın [39] showed that children whose parents used screens for > 2 h/day were more likely to manage frustration effectively when device use was restricted, whereas those whose parents exceeded 4 h were more likely to respond with crying.

*Parental mental health* also emerged as a relevant contextual factor. Wu et al. [44] found that maternal depression moderated the association between mobile device use and socioemotional outcomes: among children of mothers with elevated depressive symptoms, screen use exceeding one hour per day was associated with higher levels of sleep (*β* = 9.87, 95% CI [1.34, 18.40]) and attention problems (*β* = 7.20, 95% CI [1.50, 12.91]), whereas associations were weaker or non‐significant among children of mothers with lower depression levels. Niiranen et al. [43] similarly reported persistent psychosocial symptoms in children with high screen time after controlling for mental health and parenting style. Moderation analyses showed significant interactions between screen time and maternal depression or stress for several psychosocial outcomes, although only maternal stress remained significant in adjusted models. Danet et al. [32] found no moderating effects of parental stress or household chaos on executive functioning but reported that one third of parents used mobile devices to manage children's tantrums, a practice associated with weaker executive functioning (aOR = 1.29, 95% CI [1.01, 1.65], *p* < 0.05) and poorer inhibitory self‐control (aOR = 1.24, 95% CI [0.97, 1.57], *p* < 0.05).

*Shared screen use* did not consistently mitigate negative developmental outcomes. Gath et al. [46] reported negative associations between screen‐based activities and language production and comprehension. In contrast, Cadime et al. [47] found that frequent shared book reading without screens was positively associated with expressive vocabulary, while higher daily screen exposure was inversely related to shared reading frequency. Mallawaarachchi et al. [33] noted that children spent the largest proportion of mobile screen time co‐using with parents, although co‐use was not significantly associated with internalising or externalising behaviours. However, Rayce et al. [35] showed that language comprehension difficulties associated with screen use exceeding 1 h per day were amplified in the absence of frequent shared reading. Similarly, Wannapaschaiyong et al. [37] found that unsupervised device use, absence of screen‐time limits and exposure to low‐quality content were strongly associated with executive function impairments. Conversely, Wu et al. [44] highlighted the protective role of shared reading for socioemotional outcomes, while Mustonen et al. [41] reported a positive, though non‐significant, association between shared screen time and language development.

### 5.4 Impact of Device Use on Parent–Child Interactions and Closeness

The impact of screen use on family relationships and parent–child interactions was also addressed by several studies. Gath et al. [46] found that prolonged use of television and electronic devices was associated with lower parent–child closeness (*r* = −0.29 and *r* = −0.37, *p* < 0.01) and poorer quality of family interactions involving storytelling, sustained conversations or teaching new words (*r* = −0.32, *p* < 0.01 and *r* = −0.27, *p* < 0.05, respectively). Television viewing negatively affected parent–child emotional closeness indirectly through reduced interaction quality (*β* = −0.28, *p* = 0.04), whereas electronic device use showed a direct negative effect on emotional closeness without mediation (*β* = −0.26, *p* < 0.01). Similarly, Liu et al. [40] reported that higher media use was associated with parent–parent–child closeness (*β* = −0.15, *p* < 0.001) and poorer emotion regulation (*β* = −0.19, p < 0.001), which were in turn positively related to prosocial behaviour (*β* = 0.19, *p* < 0.05 and *β* = 0.66, *p* < 0.001), with significant independent and sequential mediation effects (e.g., chain‐mediated effect = −0.07, 95% CI [−0.12, −0.03], *p* < 0.05). Liszkai‐Peres et al. [36] observed that 65.3% of preschool children used mobile touchscreen devices (MTSDs) for at least 2 h/week. Regular MTSD users spent significantly less screen‐free leisure time with parents compared with non‐users (2.82 h/day vs. 3.94 h/day, *p* < 0.01) and showed lower parent–child interactivity (*β* = 0.08, *p* = 0.006). Shared offline activities were negatively associated with MTSD‐related conflict (*β* = −1.77, *p* = 0.009) and children's total media consumption correlated strongly with parental use (*r* = 0.63, *p* < 0.001). Conversely, Schwarzer et al. [45] found that higher levels of parent–child interaction were significantly associated with better motor (*b* = 0.43, 95% CI [0.03–0.84], *p* = 0.04), cognitive (*b* = 0.52, 95% CI [0.09–0.95], *p* = 0.02), language (*b* = 0.42, 95% CI [0.02–0.83], *p* = 0.04) and socioemotional development (*b* = 0.77, 95% CI [0.35–1.19], *p* < 0.001).

## 6 Discussion

This systematic review aimed to update the literature on the effects of excessive screen use during early childhood and the moderating role of families in this relationship. Overall, the findings corroborate significant associations between screen time and multiple developmental domains, as well as the influence of family factors on the magnitude and direction of these effects. Most of the included studies employed cross‐sectional designs, limiting conclusions about the stability of early screen use patterns and their long‐term developmental consequences [33]. Nevertheless, a consistently high proportion of studies reported screen use exceeding paediatric recommendations [2], confirming a generalised trend of noncompliance also observed in previous research [50].

Research interest has focused primarily on socioemotional development, in line with earlier systematic reviews [1, 30, 31]. Screen use in early childhood was consistently associated with poorer social and socioemotional outcomes, including reduced social skills, emotional regulation difficulties, impulsivity, emotional lability, aggressiveness, low frustration tolerance and anxiety. These findings align with prior evidence suggesting that using digital media to calm children may increase negative emotionality over time by disrupting social interactions, limiting opportunities for self‐regulation learning and reinforcing associations between media use and immediate reward [20, 21].

Regarding cognitive development, excessive screen use was generally associated with poorer executive functioning [30, 51, 52]. However, the absence of this association in the two longitudinal studies raises questions about potential child‐driven effects and the moderating role of developmental stage and screen use characteristics. Danet et al. [32] suggest that greater screen use may reflect parental responses to pre‐existing regulatory difficulties rather than being their primary cause. Mallawaarachchi et al. [33] further argue that associations between screen use, cognition and behaviour are small and vary by age and purpose of use, emphasising distinctions between passive viewing and interactive use. While some studies report weak positive associations between educational content and developmental outcomes [34, 42], these findings are consistent with evidence indicating benefits only when recommended screen time limits are respected [51]. Overall, the evidence remains inconsistent, with small effect sizes and substantial methodological heterogeneity, as also highlighted in earlier reviews [29, 53].

Language development was negatively associated with daily screen exposure exceeding 1 h, particularly in relation to comprehension, production and expressive vocabulary. Excessive screen use may reduce opportunities for language learning through social interaction with caregivers and peers [30, 54, 55]. However, high‐quality educational content may support language development when screen time recommendations are followed, consistent with previous reviews [51, 54].

Evidence regarding physical and motor development was limited and inconsistent. Some studies reported associations between higher screen use and poorer gross motor development, whereas others found no such association and even reported positive links with fine motor skills, possibly reflecting practice of manual gestures transferable to other learning contexts [42, 56]. Research focusing specifically on physical development was scarce, with greater attention given to sleep problems associated with sedentary screen use, suggesting sleep disruption as a potential pathway linking excessive screen exposure to other developmental outcomes [30, 53].

Family involvement emerged as a central moderating factor in the relationship between screen use and child development. Active parental mediation, including supervision, co‐viewing and guided use, was consistently associated with reduced negative effects of screen exposure [54]. When adults actively engage with children around screen content, digital media may support learning rather than replace direct interaction [54, 55]. However, parental involvement is not uniformly protective. Higher parental screen use was consistently associated with greater child exposure [39, 41, 45, 48], a pattern supported by previous reviews and meta‐analyses [24, 25]. In this context, parental screen use may act as a risk factor, particularly when devices are used to manage children's behaviour or emotions [20]. These risks appear amplified in families experiencing parental stress or depressive symptoms, which have been linked to increased child screen use and adverse outcomes in sleep, attention and behaviour [57, 58].

In sum, the results obtained from this systematic review provide an updated and comprehensive overview of the relationship between screen time, child development and parental involvement. Nevertheless, several limitations should be taken into account when interpreting these findings. First, most of the included studies employ a cross‐sectional design, which limits the ability to establish causal relationships between digital device use and its effects on development. This methodological limitation restricts the possibility of understanding how these effects evolve over time. Therefore, a potential direction for future research lies in the development of longitudinal studies that allow for the analysis of changes in the effects of screen use over time, with the aim of establishing more robust conclusions regarding this issue. Second, a wide variety of instruments has been observed to measure both the amount and type of screen use, as well as developmental dimensions and family involvement. This diversity hinders direct comparisons across studies and may affect the consistency of the results obtained. In addition, there is a limited representation of studies focusing on children aged 6 and 7 years, indicating greater research emphasis on the effects experienced by children aged 2–5 years. This limitation reduces the ability to generalise the findings across the full age range considered in this review. In future studies, efforts should be made to homogenise screen time measurement instruments to facilitate comparisons across studies and improve the consistency of findings. Finally, a limited number of articles specifically examine the physical and motor dimensions of child development, which prevents the establishment of solid conclusions in this area and highlights the need for future research that explores this domain in greater depth, paying particular attention to motor development, sleep habits and other related aspects of this developmental domain. Lastly, it would be advisable to design educational intervention programmes aimed at parents and caregivers to promote healthy and conscious use of technology in the home and to investigate their effectiveness in children's development.

## 7 Conclusions and Practical Implications

The findings of the present study invite consideration of the need to adopt a critical perspective on screen use, given its implications for cognitive, language, physical and motor and socioemotional development. Although protective factors for child development have been identified in relation to screen use, such as the consumption of high‐quality educational content and parental practices of co‐viewing and shared reading, the effects of these protective factors do not appear to be sufficiently strong to counterbalance the evidence supporting the need to limit screen time and to promote parental practices of active mediation, usage restriction, supervision and control of consumed content in order to mitigate the potential negative effects of screen use in early childhood and later development. Families play a key role in managing and moderating children's device use, acting either as a protective or a risk factor depending on the practices they adopt. Parental modelling, active supervision, caregivers' mental and emotional well‐being and the quality of the affective bond are associated with more favourable child development and with moderation of the adverse effects of excessive screen use. Therefore, clear rules should be established and children should be accompanied during digital media use; parental well‐being should be considered a key element in the prevention of problematic screen use in childhood; conscious, healthy and consistent digital habits should be promoted among adults; and parent–child interactions outside of screen use should be strengthened.

In conclusion, this review underscores the need to promote moderate, conscious and supervised use of digital devices during early childhood, in line with the recommendations of health and paediatric organisations. Families and educators should develop critical awareness of both the benefits and risks associated with screens and excessive technology use. Educational efforts should promote healthy digital competence from early ages, while also strengthening the affective bond through shared activities that do not rely exclusively on technology. Only through active and coherent involvement by the adult environment will it be possible to ensure adequate holistic development and a healthy relationship with digital technologies.

## Funding

Funding for open access charge: Universidade da Coruña/CISUG (Case Number: 28381428).

## Ethics Statement

Ethical approval was not required for this study as it is based exclusively on the analysis of previously published literature. The authors declare that the research presented in this manuscript complies with the ethical principles established by the University of A Coruña. No human subjects were involved, as this is a systematic review.

## Consent

The authors have nothing to report.

## Conflicts of Interest

The authors declare no conflicts of interest.

## Supporting information


**Table S1:** Risk of bias in the studies included in the review on screen use and child development.
**Table S2:** Characteristics of the studies included in the review on screen use and child development.

**Data S1:** jpc70533‐sup‐0002‐PRISMA_2020_checklist_1.docx.

## Data Availability

No new data were generated or analysed in this study. All data analysed are available in publicly accessible repositories and published sources cited in the manuscript. Study materials and methodological documentation used in this systematic review are available in the Open Science Framework: https://osf.io/35mtz/overview.
