# Supplementary material for: Screen Use, Child Development and Family Involvement in Children Aged 2–7 Years: A Systematic Review
Source: J Paediatr Child Health. 2026 Aug 11;62(10):1921–30. doi: 10.1111/jpc.70533 (PMC13671253; doi:10.1111/jpc.70533)
Supplement: Supplementary file 2 [file JPC-62-1921-s002.docx]

**Table 1. Supplementary material**

*Risk of bias in the studies included in the review on screen use and child development*

|  |  |  |  | **Reviewer 1** | | | | | | | | **Reviewer 2** | | | | | | | | **Final decision** | |
| --- | --- | --- | --- | --- | --- | --- | --- | --- | --- | --- | --- | --- | --- | --- | --- | --- | --- | --- | --- | --- | --- |
| **Author/s** | **Year** | **Design** | **Sample** | **I1** | **I2** | **I3** | **I4** | **I5** | **I6** | **I7** | **I8** | **I1** | **I2** | **I3** | **I4** | **I5** | **I6** | **I7** | **I8** | **Risk of bias** | **Main justification** |
| Cadime et al. | 2025 | Cross-sectional | *n =* 362 | Y | Y | Y | U | U | Y | Y | Y | Y | Y | Y | U | U | Y | Y | Y | Moderate | Adequate sample size and use of validated instruments; however, screen exposure was measured through parent-reported questionnaires. |
| Chaibal & Chaiyakul | 2022 | Cross-sectional | *n* = 85 | Y | Y | U | U | N | Y | Y | Y | Y | Y | U | U | N | Y | Y | Y | Moderate | Small sample size and device use measured through caregiver self-report. |
| Danet et al. | 2022 | Longitudinal | *n* = 368 | Y | Y | Y | Y | Y | Y | Y | Y | Y | Y | Y | Y | Y | Y | Y | Y | Low | Longitudinal design and use of validated scales to assess executive functioning. |
| Gath et al. | 2023 | Cross-sectional | *n* = 84 | Y | Y | Y | U | U | Y | Y | Y | Y | Y | Y | U | U | Y | Y | Y | Moderate | Small sample size, although standardized language assessments were used. |
| John et al. | 2021 | Cross-sectional | *n* = 189 | Y | Y | U | U | U | Y | Y | Y | Y | Y | U | U | U | Y | Y | Y | Moderate | Reliance on parental questionnaires and correlational analysis. |
| Liszkai-Peres et al. | 2024 | Cross-sectional | *n* = 72 | Y | Y | U | U | N | Y | Y | Y | Y | Y | U | U | N | Y | Y | Y | Moderate | Small sample and parent-reported interaction measures. |
| Liu et al. | 2024 | Cross-sectional | *n* = 701 | Y | Y | Y | Y | Y | Y | Y | Y | Y | Y | Y | Y | Y | Y | Y | Y | Low | Large sample and use of validated socioemotional development scales. |
| Mallawaarachchi et al. | 2025 | Longitudinal | *n =* 536 | Y | Y | Y | Y | Y | Y | Y | Y | Y | Y | Y | Y | Y | Y | Y | Y | Low | Longitudinal design and standardized behavioral assessment instruments. |
| Mustonen et al. | 2022 | Cross-sectional | *n =* 164 | Y | Y | Y | U | U | Y | Y | Y | Y | Y | Y | U | U | Y | Y | Y | Moderate | Standardized language assessments were used, but screen exposure was self-reported. |
| Niiranen et al. | 2024 | Cross-sectional | *n* = 671 | Y | Y | Y | Y | Y | Y | Y | Y | Y | Y | Y | Y | Y | Y | Y | Y | Low | Large sample and statistical control of parental mental health and parenting variables. |
| Nobre et al. | 2020 | Cross-sectional | *n* = 103 | Y | Y | Y | U | U | Y | Y | Y | Y | Y | Y | U | U | Y | Y | Y | Moderate | Use of Bayley-III developmental assessment, although sample size was relatively small. |
| Oflu et al. | 2021 | Cross-sectional | *n* = 240 | Y | Y | U | U | U | Y | Y | Y | Y | Y | U | U | U | Y | Y | Y | Moderate | Screen use and supervision were measured through parental surveys. |
| Rai et al. | 2023 | Cross-sectional | *n* = 44 | Y | U | U | N | N | Y | U | Y | Y | U | U | N | N | Y | U | Y | High | Very small sample size and reliance on parental diary reports. |
| Rayce et al. | 2024 | Cross-sectional | *N* = 31.125 | Y | Y | Y | Y | Y | Y | Y | Y | Y | Y | Y | Y | Y | Y | Y | Y | Low | Very large sample size and use of standardized developmental questionnaires. |
| Sari & Yalçin. | 2024 | Cross-sectional | *n* = 464 | Y | Y | U | U | U | Y | Y | Y | Y | Y | U | U | U | Y | Y | Y | Moderate | Screen use assessed through questionnaires and correlational analysis. |
| Schwarzer et al. | 2022 | Cross-sectional | *n* = 296 | Y | Y | Y | U | U | Y | Y | Y | Y | Y | Y | U | U | Y | Y | Y | Moderate | Use of standardized developmental tests, although exposure data were parent-reported. |
| Wannapas-Chaiyong et al. | 2023 | Cross-sectional | *n* = 110 | Y | Y | Y | U | U | Y | Y | Y | Y | Y | Y | U | U | Y | Y | Y | Moderate | Validated executive functioning scales but relatively small sample size. |
| Wu et al. | 2023 | Cross-sectional | *n* = 202 | Y | Y | Y | U | U | Y | Y | Y | Y | Y | Y | U | U | Y | Y | Y | Moderate | Validated behavioral measures but reliance on parent-reported screen exposure. |

Note: I – Items: (1) inclusion criteria clearly defined, (2) study subjects and setting described, (3) exposure measured validity and reliably, (4) objective criteria for condition measurement, (5) confounding factors identified, (6) strategies to deal with confounding factors, (7) outcomes measured validly and reliably, (8) appropriate statistical analysis used; Y – Yes, N – No, U – Unclear, NA – Not applicable

**Table 2. Supplementary material**

*Characteristics of the studies included in the review on screen use and child development*

| **Author/s** | **Year** | **Country** | **Design** | **Sample** | **Dependent variables** | **Instruments** | **Key findings** |
| --- | --- | --- | --- | --- | --- | --- | --- |
| Cadime et al. | 2025 | Portugal | Cross-sectional design | *n =* 362  Children aged 2.5-3.5 years | Language development | - MacArthur-Bates CDI - Parent-child shared reading (1 item) - Screen exposure questionnaire. - Video-call frequency questionary - Sleeping problems scale | - Children’s average screen time was 3.23 hours/day (SD = 233) - Children’s screen exposure was negatively associated with shared book reading and vocabulary development, and video call frequency was positively associated with sleep problems. - Screen exposure and video call frequency were not associated with syntactic development. - Shared book reading was the strongest predictor of children’s language outcomes. - Higher maternal education was associated with lower screen exposure and higher frequency of shared reading, indirectly supporting language development. |
| Chaibal & Chaiyakul | 2022 | Thailand | Cross-sectional design | *n* = 85  Children aged 2-5 years | Language, motor, and socioemotional development | - Questionnaire to collect descriptive data, socioeconomic demographic and smartphone and tablet usage parameters, from caregivers. - Denver Developmental Screening Test II (Denver II). | - Children spend an average of 82.78 minutes per day using smartphones and tablets (SD = 62.82). - Children’s device use was significantly correlated with the amount of time their mothers and other relatives spent using digital devices. - An association was observed between children’s smartphone and tablet use and gross motor development. |
| Danet et al. | 2022 | USA | Longitudinal design | *n* = 368  Children aged 3-4 years | Cognitive development (executive functioning) | - Parenting Stress Index-Short Form (PSI-SF). - Confusion, Hubbub and Order Scale (CHAOS). - Behavior Rating Inventory of Executive Function–Preschool (BRIEF-P). - Child Mobile Device Use. | - 50% of the children used digital devices for more than 1 hour/day and 1 out of 3 children used devices to calm down, and 39.7% as educative apps, presented lower executive functioning. - Children with weaker executive functioning were more likely to be given mobile devices to calm them. - Screen-based activities (video watching, use of age-inappropriate applications, or device use exceeding 1 hour/day) were not associated with executive functioning. - Parenting stress and household chaos did not moderate these associations. |
| Gath et al. | 2023 | New Zeeland | Cross-sectional design | *n* = 84  children aged 3-5 years | Language and socioemotional development | - Child screen time report. - Quality of parent-child interactions scale. - Questionnaire on shared reading within the home. - Recalling Sentences subscale of the Clinical Evaluation of Language Fundamentals Preschool (CELF-P2). - Story recall task. - Child-Parent Relationship Scale (CPRS) | - Children’s average screen time was 1.78 hours/day on weekdays and 2.65 hours/day on weekends. - Higher screen time was associated with lower language production and comprehension scores. - Higher screen time was associated with lower levels of parent–child closeness. - Screen time showed indirect effects on language production and comprehension, and parent–child closeness, through reduced shared reading and poorer quality of family interactions. |
| John et al. | 2021 | India | Cross-sectional design | *n* = 189  children aged 2-5 years | Cognitive and socioemotional development | - Self-administered questionnaire on parental practices regarding child screen viewing. - Werner David Development pictorial scale (WDDPS). | - 89.9% of children exceeded recommended screen time, averaging 2.14 h/day; screen time and supervision were not associated with child or maternal sociodemographic variables. - Mealtime viewing and on-demand screen use were associated with excess screen time. - Risk factors for excess screen time included non-computer device use, mealtime, and on-demand viewing. - Inconsistent parental supervision was associated with non-computer device use, unplanned viewing, and lack of co-viewing. - Excess screen time was not associated with developmental outcomes, whereas inconsistent supervision was associated with lower physical activity and suspected cognitive delays. |
| Liszkai-Peres et al. | 2024 | Hungary | Cross-sectional design | *n* = 72  Children aged 4-7 years | Socioemotional development | - Digital Media Use Questionnaire (DMUQ). - Parent–child interaction test (PCIT). | - Parents in the non-user group were more educated than those in the mobile touchscreen devices (MTSD)-user group, with no differences in family income. - Shared online activities and higher parental education predicted lower parent–child interactivity; meanwhile, shared offline activities predicted lower MTSD conflict and lower problematic MTSD. - Higher parental education and greater child media consumption were associated with higher levels of problematic MTSD use. - Higher parental education and greater child media consumption independently predicted problematic MTSD use. - Children’s total media use was strongly correlated with parental media use, particularly TV viewing. |
| Liu et al. | 2024 | China | Cross-sectional design | *n* = 701  Children mean age of 5.20 years (SD = 1.87) | Socioemotional development | - Children’s Electronic Media Use Questionnaire (adapted) - Child–Parent Relationship Scale (adapted) - Emotion Regulation Checklist. - The Strengths and Difficulties Questionnaire (SDQ). | - Children’s use of electronic media was medium-low (M = 2.35). - Children’s use of electronic devices negatively correlated with prosocial behavior, parent-child closeness, and emotion regulation. - The association between parent-child closeness and emotion regulation mediated the relationship between electronic media use and prosocial behavior. |
| Mallawaarachchi et al. | 2025 | Australia | Longitudinal design | *n =* 536  Children aged 1-6 years | Socioemotional development | - Mobile screen use questionnaire - BITSEA - SQD | - 66.4% of children used mobile screens an average of 33 min/day (SD = 50 min), especially to watch entertainment programs or videos (56%) and co-using the mobile phone with parents (46%). The usage patterns were stable over one year. - No cross-sectional associations were found between mobile device use (type or social context) and internalizing or externalizing behaviors. - Longitudinally, in toddlers, a lower proportion of program viewing (i.e., greater interactive app use) predicted higher externalizing behaviors at follow-up (*β* = -.42). No significant longitudinal associations were observed for preschool-aged children. |
| Mustonen et al. | 2022 | Finland | Cross-sectional design | *n =* 164  Children mean age of 2.5-4.1 years | Language development | - The Screen Time Questionnaire (STQ). - Finnish version of MacArthur Communicative Development Inventories III (FinCDI III Words). - Finnish Phonology test (FPT). - Finnish Morphology test (FMT). - Reynell Developmental Language Scales III (RDLS III). | - Children used screens for an average of 79 min/day, including 44 min/day of unsupervised use and 34 min/day of accompanied use. - Greater screen time in both children and mothers was associated with weaker lexical and overall language abilities in children. - When both factors (child and maternal screen time) were included in the analysis, a cumulative negative effect on children’s lexical and overall language skills were observed. |
| Niiranen et al. | 2024 | Finland | Cross-sectional design | *n* = 671  Children aged 5 years | Cognitive and socioemotional development | - E-media use questionnaires. - The Center for Epidemiologic Studies Depression Scale (CES-D scale). - Perceived Stress Scale (adapted). - Finnish version of Block’s Child Rearing Practices Report (CRPR). - Five-to-Fifteen questionnaire (FTF). | - 68.8% of children use screens for an average exceeding 60 minutes per day. - High levels of screen time in 5-year-old children were associated with greater difficulties in attention, concentration, hyperactivity, impulsivity, as well as internalizing and externalizing symptoms. These associations remained significant after controlling parental mental health and parenting style. - Maternal stress and depression moderate the association between screen time and children’s psychosocial symptoms. |
| Nobre et al. | 2020 | Brasil | Cross-sectional design | *n* = 103  Children mean age of 2-3.5 years | Cognitive, language and motor development | - Questionnaire on habits related to the use of interactive means. - Brazilian Criteria of Economic Classification (CCEB). - Third edition of the Bayley Scale of Infant and Toddler Development (Bayley-III) | - Daily use of electronic devices was less than one hour. However, usage ranged from a minimum of 10 min/day to a maximum of 480 minutes. - A positive association was found between the quality of media use and language development, along with weaker correlations with cognitive development and fine motor skills. - The multicriteria index developed explained 20% of the variance in language performance but was not significant for cognitive or fine motor domains. |
| Oflu et al. | 2021 | Turkey | Cross-sectional design | *n* = 240  Children ages 2-5 years | Socioemotional development | - Structured survey on daily screen time. - Emotion Regulation Checklist (ERC). | - 40,8 % of children had screen use of 4h/day. This level of use was associated with having started screen exposure at 12 months of age or later, being primarily cared for by the mother, and the absence of parental supervision. - Children with ≥ 4 hours of screen use and no parental supervision showed greater emotional lability/negativity. High screen use was more likely among children primarily cared for by their mothers and those who did not engage in co-viewing with parents. |
| Rai et al. | 2023 | Canada | Cross-sectional design | *n* = 44  Children aged 3 years | Cognitive and language development | - 2-week online daily diary survey about screen use patterns in children. - Four tests to assess cognitive development: expressive vocabulary; head, toe, knees, and shoulders (HTKS); word range; and snack delay. - Three tasks to measure parent-child interactions coded using the Parent–Child Interaction System (PARCHISY). | - Children’s mean daily screen use was 103.5 minutes (SD = 59.2). Of this time, 24.9 minutes (SD = 29.5) corresponded to unsupervised mobile phone use, and 48.1 minutes (SD = 30.5) were spent using devices accompanied by parents. - Educational screen time was positively associated with cognitive development, whereas other types of content (watching programs, movies, or videos) and joint media use were negatively associated with cognitive development . - Medium to large effect sizes were observed for differences in the quality of parent–child interaction across the three tasks . |
| Rayce et al. | 2024 | Denmark | Cross-sectional design | *N* = 31.125  Children aged 2-3 years | Language development | - Child mobile device screen time. - Five to Fifteen Toddlers questionnaire (FTF-Toddlers) - Sociodemographic questionnaire - WHO-5 Parental well-being. | - 72.2% of children use mobile phones: 62.2% use them for less than 1 h/day and 10% use them for 1 h/day. - 88% of children use television or computers (75.5% use them for 1 h/day or less) - Screen time of 1 h/day or more was associated with poorer language development and higher odds of difficulties in language comprehension and expressive language skills. - Frequent shared reading mitigated the negative effect on language comprehension, but not on expressive language abilities. - No moderating effects were observed for parental educational level or for time children spent watching television or using computers. |
| Sari & Yalçin. | 2024 | Turkey | Cross-sectional design | *n* = 464  Children aged 3-4 years | Socioemotional development | - General characteristics of father-child pairs questionnaire. - Media usage characteristics questionnaire. - UNICEF Early Childhood Development Index (ECDI). | - 50.6% of the children used digital devices for 2 h/day or more, and 41.8% began using screens 12 months of age or earlier. Age-appropriate content was consumed by 88.4% of the children. - 50% of children use devices during mealtimes. Mobile devices were used as a reward (35.3%) and as a punishment (38.1%). - Overall, 89.7% of children showed adequate development in at least three of the four domains of the Early Childhood Development Index (ECDI). - When screen use was restricted, 55.6% of children engaged in another activity, whereas 44.4% reacted by crying. - Children’s reactions to screen restriction were associated with both child and paternal screen time, the presence of three or more books at home, quality of care, and good performance in literacy and numeracy. |
| Schwarzer et al. | 2022 | Germany | Cross-sectional design | *n* = 296  Children aged 2-5 years | Cognitive, language, socioemotional and motor development | - German Health Interview and Examination Survey for Children and Adolescents (KiGGS). - Mütterfragebogen zu kindlichen Aktivitäten im Kontext des Familiensettings (AKFRA). - Development Test 6 Months to 6 Years - revised (ET 6-6-R). | - The average daily device use was 0.75 hours (SD = 0.7), with 24% of children using devices for 1 h/day or more. - Mean daily television viewing time was 0.55 hours (SD = 0.56). - High screen times in children (>1 h/day) were associated with lower percentile ranks in cognition, language, and social–emotional skills. - High screen times in mothers (>5 h/day) were associated with high media use by children. - Higher parent–child interaction scores were significantly associated with better body motor, cognition, language, and social–emotional outcomes in children. |
| Wannapas-Chaiyong et al. | 2023 | Thailand | Cross-sectional design | *n* = 110  Children aged 4-6 years | Cognitive and socioemotional development | - Sociodemographic information form - Parenting Style and Dimensions Questionnaire – Short version (PSDQ) - Behavior Ration Inventory of Executive Function- Preschool version. | - The average device use was 2 h/day, of which 1.75 hours were reported as high-quality content. 52% of children spend more than 50% of their screen time under adult supervision. - Executive dysfunction was identified in 23.6% of the children. - Unsupervised device use in children was associated with executive dysfunction, particularly when children had co-viewing with caregivers for less than 50% of total screen time. - Less co-viewing was linked to specific executive function deficits: inhibition, emotional control, planning and organization. - Extended exposure to low-quality media content was associated with impaired working memory. |
| Wu et al. | 2023 | Taiwan | Cross-sectional design | *n* = 202  Children aged 2-5 years | Cognitive, socioemotional, and motor (physical) development | - Child Behavior Checklist (CBCL). - Questionnaire on mobile devices used by children and mothers. - Structured questionnaire about parent-child shared reading. - Edinburgh Postnatal Depression Scale (EPDS). | - Mobile devices were used by 68.5% of children. Among them, 79.9% used devices for less than 1 h/day, whereas 20.3% exceeded this threshold. - Children who used devices for less than 1 h/day (mean = 20.75 min/day, SD = 14.09) were more likely to engage in shared reading (95.8%) compared to those with excessive use (M = 102 min/day, SD = 50.07), among whom only 76.7% participated in shared reading. - Mothers’ higher usage time on mobile devices and an education level of college or less were associated with the child’s exceeding recommended use of mobile devices. - Among depressed mothers, preschoolers’ exceeding recommended use of mobile devices was associated with more sleep and attention problems, whereas shared reading was associated with less somatic complaints and withdrawn, compared with their respective counterparts. |

*Note.* Own elaboration.
